# Development of a multi-wavelength diffuse optical tomography system for early diagnosis of rheumatoid arthritis: simulation, phantoms and healthy human studies

**DOI:** 10.1364/BOE.7.004769

**Published:** 2016-10-27

**Authors:** Hao Yang Wu, Andrew Filer, Iain Styles, Hamid Dehghani

**Affiliations:** 1 The Centre for Physical Sciences of Imaging in Biomedical Sciences (PSIBS), University of Birmingham, Edgbaston, Birmingham, B15 2TT, UK; 2 The Rheumatology Research Group, School of Immunity and Infection, College of Medical and Dental Sciences, University of Birmingham, Edgbaston, Birmingham, B15 2TT, UK; 3 School of Computer Science, University of Birmingham, Edgbaston, Birmingham, B15 2TT, UK

**Keywords:** (120.4820) Optical systems, (170.1470) Blood or tissue constituent monitoring, (110.6955) Tomographic imaging

## Abstract

A multi-wavelength diffuse optical tomography (DOT) system has been developed to directly extract physiological information, such as total haemoglobin concentration, from tissue in human hand joints. Novel methods for 3D surface imaging and spectrally constrained image reconstruction are introduced and their potential application to imaging of rheumatoid arthritis is discussed. Results are presented from simulation studies as well as experiments using phantoms and data from imaging of three healthy volunteers. The image features are recovered partially for phantom data using transmission measurements only. Images that reveal joint regions and surrounding features within the hand are shown to co-register with co–acquired ultrasound images which are shown to be related to total haemoglobin concentration.

## 1. Introduction

Rheumatoid arthritis (RA) is a persistent autoimmune disease causing impairment of multiple joints, of which the hand joints are most commonly affected [1]. Early diagnosis and intervention is a key step in order to arrest disease progression and facilitate optimal treatment outcome. The underlying pathological changes in the joint due to RA in early disease have attracted interest in recent years [2, 3]. The characteristics of RA disease are known to include localised inflammation in the synovial lining of the joint, in which the synovium becomes hyperplastic, and is infiltrated by numerous immune cells and expansion of existing stromal cell populations. Following the cell proliferation, the alternation of physical appearances in the joint includes the turning of clear fluid to greyish turbid media, and the narrowing of joint spaces. Subsequently, the activation of bone degradation pathways leads to bone erosion [1]. The end result of RA disease course is severe hand deformity and functional disability. The elucidation of synovial disease pathways suggests that hypoxia and the elevation of oxygenation levels to meet the demands of the tissues, mediates new vasculature formation (angiogenesis). These are key events in the early stages of RA pathology [4,5]. Vascular flow as measured using MRI and ultrasound has been shown to correlate with the level of bone erosion and angiogenesis within the synovium [6, 7]. Optical Near Infrared (NIR) techniques have shown promise in the clinic for early diagnosis of RA and potential to overcome the limitations such as high cost, the necessity for contrast agents, ionising radiation, long acquisition time, limited accessibility to small joints, tedious examination procedures and substantial difficulty of data interpretation, as well as the semi–quantitative nature of the analysis [8, 9], of existing imaging modalities, including MRI, ultrasound, and CT.

Several studies have explored uses of NIR techniques and systems to characterise disease in the joint, including RA. One such example included the use of a NIR imaging system using a single wavelength laser diode for continuous–wave (CW) illumination, and a CCD camera for detection [10]. The system captured trans-illumination images of finger joints, and utilized them directly to classify inflamed joints from non–inflamed ones. The sensitivity and specificity was reported in the range of 80% - 90% with clinical examination as the reference, which was achieved using machine–learning methods [10]. More recently, NIR tomographic imaging was introduced, and a reconstruction algorithm solving the full radiative transfer equation (RTE) was used to retrieve a 3D image volume from measurements [12]. The system operated in scanning mode by integrating a translation stage for the source and detector to obtain multiple measurements to enhance the localisation accuracy and the reconstruction quality. The source was a single wavelength laser diode but the intensity was modulated to primarily prevent interference due to ambient light, which could also be achieved by imaging in light-tight conditions. Changing the detector to a silicon photodiode instead of a CCD camera allowed for easier translation for multiple illumination positions. The reconstructed tomographic images showed elevated scattering and absorption coefficients in the area of the joint cavity for arthritic joints as compared to the same location for healthy joints, and results agreed well with clinical and ultrasound examination which together diagnosed tenderness, swelling, and effusion within joints, confirmed by data from imaging six proximal interphalangeal (PIP) finger joints from two patients during two visits [9]. With a larger group of subjects, sensitivity and specificity of 70% was reported [11].

Optical tomography based techniques for imaging RA joints have been modified recently to improve sensitivity towards clinically useful levels. Along with the light modulation presented in the previous system, a detection modulation using an image intensifier coupled to a CCD camera was introduced allowing both phase and amplitude to be acquired. This was introduced to improve the separation of scattering and absorption coefficients, and the reduction in crosstalk of these two optical properties was shown to improve the achievable sensitivity and specificity. At the same time, a laser line-scanning unit was installed to probe the shape of the finger geometry for the creation of a model needed for tomographic reconstruction. The results demonstrated a sensitivity of 81% and a specificity of 78% when compared to clinical examination and ultrasound and magnetic resonance imaging used as references [13]. A sensitivity and specificity of above 90% was achieved when multiple image features based on both absorption and scattering coefficients were combined with computer - aided diagnosis [14]. In another study, a new system was built in which data at two wavelengths were measured from which tissue oxygenation was quantified [15]. The reconstructed images of total haemoglobin concentration (deoxygenated and oxygenated concentration) showed increased vascular flow around the RA joint, and this was also observed with other studies confirming a rise in oxygen consumption and metabolism. The anatomical features in the reconstructed images were consistent with those from the single wavelength measurement based reconstruction. This system had a cylindrical source–detector array that was limited to imaging only proximal interpphangeal (PIP) joints. Finally, to address cellular specificity, a system was developed to image a 2D distribution of fluorescence from indocyanine green (ICG) injected into the body, and elevated ICG concentrations were found at hand joints affected with inflammation. Only a moderate sensitivity and specificity were shown [16].

The dual wavelength imaging system discussed above which shows the capability of quantifying tissue oxygenation is promising. Hypoxia and angiogenesis in the joint is a topical subject, and is more indicative of pathology and a potential sensitivity biomarker than the measured changes of absorption and scattering coefficients (at a single wavelength) as a result of perturbation of the joint fluid. In this work, a multi-wavelength diffuse optical tomography (DOT) imaging system is presented to directly extract functional information relating to the total haemoglobin concentration in the hand joints. The developed system has a number of modifications over other systems to allow further improvement in quantitative accuracy: (1) a spectrally constrained reconstruction algorithm is used to incorporate data from multiple wavelengths simultaneously to reduce the ill-posed nature of the parameter recovery algorithm seen in single wavelength measurement based reconstruction [17]. (2) Instead of solving the full radiative transfer equation (RTE) for tomographic reconstruction (computational intensive), the utility of the diffusion approximation of RTE with an efficient newton-like optimization method for tomographic reconstruction is assessed [18]. (3) A continuous-wave (CW) multi-wavelength system has been developed to be cost-effective [19]. A dedicated scientific grade CCD camera, which is in a non–contact vertical geometry allowing an extended view of the hand joints, is adapted to boost the signal quality, and a stand-alone structured illumination system is integrated for fast and accurate surface geometry imaging [20]. The system is demonstrated with simulations to highlight the performance improvement at the theoretical level. The system is then demonstrated with experimental data from phantoms. Finally, healthy volunteers are imaged and the reconstructed total haemoglobin concentration maps in the joint are visualised together with co-acquisition of ultrasound images to reveal structural information.

## 2. Materials and methods

### 2.1. The system layout

**Fig. 1:**
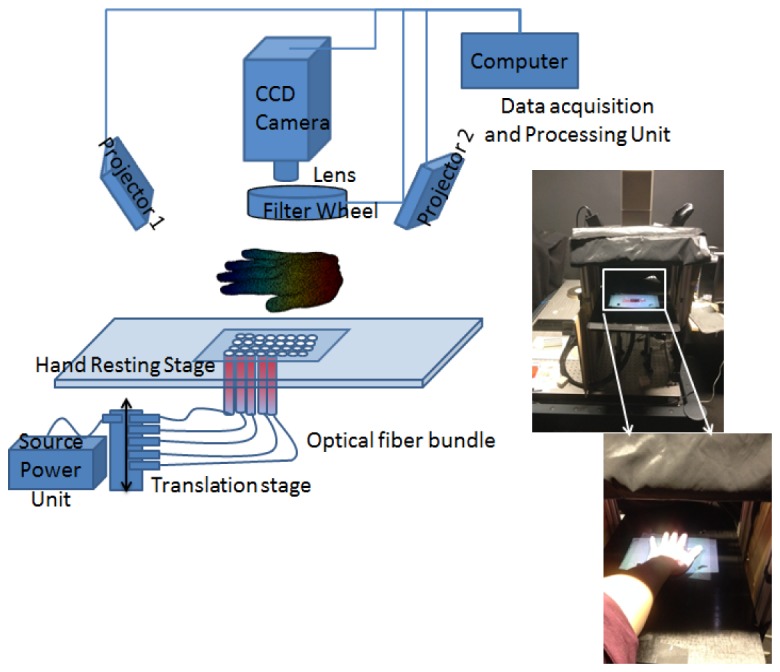
The schematic layout and image of the developed system.

The system consists of a non–contact CCD camera that is installed directly above and facing down to a resting stage, which supports the object being imaged, as shown in Fig. 1

. The camera is coupled with a 25 mm /f1.4 focal lens in the visible to near infrared spectrum range (Techspec, Edmund Optics, York, UK) with the focus adjusted to the sample stage and the aperture fully opened permitting maximum light capture. The NIR excitation is produced by a 20W tungsten – halogen lamp, which produces 8mW after passing through a 600 µm optical fibre, with the illumination spectrum ranging from 360 nm to 2400 nm (HL-2000-FHSA, OCEAN Optics, Oxford, UK). The light source is sequentially butt-coupled with 16 optical fibres (FT10 00EMT 1000 micrometre 0.39NA, Thorlabs, Ely, UK) using a translation stage (LTS150, Thorlabs, Ely, UK) to provide illuminations at multiple positions. The hand-resting stage is a black acetal sheet with holes drilled in the centre for attaching the optical fibres and mounting it on the optical bench. The light illumination passing through the object is imaged by an air-cooled back-thinned CCD camera (C4742 – 98, Hamamatsu photonic, Hamamatsu City, Japan), which features hermetic vacuum chamber technology to further reduce the thermal noise.

A filter wheel (FW102c, Thorlabs, Cambridgeshire, UK) is installed in front of the camera for decoupling spectral components of the transmitted light that enables the system to acquire images at multiple wavelengths. The filter wheel holds five bandpass interference filters (FB690-10 ~FB930-10, Thorlabs, Ely, UK) each having a 10 nm full width at half maximum. The chosen wavelengths are at 690 nm, 730 nm, 750 nm, 850 nm, and 930 nm, and are selected on the basis of their ability to separate the haemoglobin absorption spectrum from that of water [21]. Two mini pocket projectors (Mpro120, 3M, Bracknell, UK) are used to provide the structured light illumination for surface geometry imaging. All system components are mounted on aluminium posts (RS components, Corby, UK) forming an enclosure of the system. All system components are controlled by a computer (Viglen, UK), that has an Intel Core 2 quad CPU Q8200 at 2.33 GHZ and 8 GB RAM running an operating system of 64–bit Windows 7 (Microsoft, Redmond, WA), connected through standard computer ports including USB, DVI and firewire. The system control is implemented using Labview software (National Instruments, Newbury, UK). Algorithms for data manipulation and image processing are developed in MATLAB (The MathWorks, Natick, Massachusetts, United States of American). The typical data acquisition time is ~5 minutes (depending on the thickness of the object being imaged) for spectral imaging, and 40 seconds for surface geometry imaging.

For surface geometry imaging, the camera parameters were set to: high-speed readout mode (the fast acquisition); 0.02s exposure time; 1 × binning. For spectral imaging, the camera parameters are: high-precision readout mode (the high dynamic range); auto exposure to 50% of the saturation level; 2 × binning. The gain was set to high dynamic range gain as default.

### 2.2. Surface geometry imaging and mesh creation

The surface geometry imaging is based on a digital projection profilemetry that works by the sequential projection of computer-generated sinusoidal fringe patterns with multiple frequencies and phases, and the subsequent capture of the reflected and deformed image patterns. The warp phase map, ϕ, is extracted from all acquired images as follow:

$$\phi =arc\tan\frac{\sum_{i=1}^{N}\sin\left(\delta_{phaseshift}^{i}\right)I_{i}\left(u,v\right)}{\sum_{i=1}^{N}\cos\left(\delta_{phaseshift}^{i}\right)I_{i}\left(u,v\right)}.$$ (1)

Where N is the number of phase shifts at one frequency and I_i_(u,v) is the image with the fringe pattern at pixel coordinates u and v with a phase shift δ_phase shift_. The warped phase map, ϕ , at low frequency can be then used to unwarp the phase at high frequency [20]. Then the unwrapped phase map, $\Phi_{B}$, is used to calculate the surface depth of the object being imaged using [22]:

$$Z_{p}=\frac{1+C_{1}\Phi_{B}+\left(C_{2}+C_{3}\Phi_{B}\right)I_{B}+\left(C_{4}+C_{5}\Phi_{B}\right)J_{B}}{D_{0}+D_{1}\Phi_{B}+\left(D_{2}+D_{3}\Phi_{B}\right)I_{B}+\left(D_{4}+D_{5}\Phi_{B}\right)J_{B}}.$$ (2)

where I_B_ and J_B_ are the image pixel coordinates in two perpendicular directions. Z_p_ is the height of the object. C_1_ – C_5_ and D_0_ - D_5_ represent the geometry of the camera and projectors in relation to the reference plane. These parameters are predetermined constants, and are obtained through an imaging calibration procedure in which a set of objects with known heights are imaged and their height and unwrapped phase map are fitted using the linear least squares method. The calculated height map is then used to create a three-dimensional mesh that uses the mesh generator from the NIRFAST software package and the pinhole camera model [23,24].

### 2.3. Diffuse optical tomography and spectrally constrained image reconstruction

The diffuse optical tomography works in two steps. The first step is to use the forward model to predict the measurements made on the boundary given the three - dimensional model and an underlying distribution of optical property in the object. The second step is the image reconstruction that recovers the internal distribution of optical properties using the forward model together with experimentally obtained boundary data. The optical properties are assumed to correspond to these of the object when the prediction matches the experimental measured boundary data. An image reconstruction algorithm is provided with the NIRFAST software package [25].

The forward model describes the light propagation in the highly scattering medium, and is given by the diffusion approximation to the RTE as follows,

$$\left[-\nabla \kappa \left(r\right)\nabla +C_{0}\mu_{a}\left(r\right)\right]\phi \left(r\right)=q_{0}\left(r\right).$$ (3)

where $\mu_{a}$ and $\mu_{s}^{'}$ are the absorption and reduced scattering coefficients, respectively. ϕ is the isotropic photon fluence, κ is the diffusion coefficient defined as κ = 1/3($\mu_{a}$ + $\mu_{s}^{'}$). C_0_ is the speed of light and $q_{0}$ is the source term. The finite element method (FEM) using the mesh generated from surface geometry imaging data is used as a general and flexible method for solving the forward model for arbitrary geometries. The image reconstruction is a non- linear and ill-posed problem that is solved using the Levenberg - Marquardt algorithm that iteratively updates a given initial optical property, using:

$${\left(J^{T}J+RI\right)}^{-1}J^{T}\partial \phi =\partial \mu .$$ (4)

where ∂μ is the update to the optical properties, ∂ϕ is the boundary data mismatch at each iteration and J is the Jacobian matrix that relates a change in the boundary data to a small change in the optical property.*R* is the regularization parameter. The image reconstruction seeks a solution where an objective function is converged to a predefined criterion which is typically a 2% change in projection error. The objective function is given as,

$$E\left(\mu_{a},\mu_{s}^{'}\right)=Y-F{\left(\mu_{a},\mu_{s}^{'}\right)}^{2}$$ (5)

where F is the boundary data generated using the forward model, and Y is the experimentally obtained data. ${\left\|\;\right\|}^{2}$ is the L2 norm. This is sometimes referred to as the projection error.

In the spectrally constrained reconstruction, absorption and scattering coefficients are combined at multiple wavelengths using the linear dependence of chromophore (oxygenated, deoxygenated haemoglobin and water) concentrations on tissue absorptions,

$$\mu_{a}\left(\lambda \right)=\sum \varepsilon {\left(\lambda \right)}_{i}C_{i}$$ (6)

and the empirical approximation to Mie’s scattering theory,

$$\mu_{s}\left(\lambda \right)=a\lambda^{-b}.$$ (7)

where $\varepsilon_{i}$(λ) is the wavelength-dependent extinction coefficient for i*th* chromophore. $C_{i}$ is the concentration, *a* and *b* are the scattering amplitude and power respectively. The spectral Jacobian now relates a small change in chromophore concentration and scattering parameters directly to a perturbation in the boundary data simultaneously at multiple wavelengths,

$$\left(\begin{array}{c} \partial \phi_{\lambda 1}\\ \partial \phi_{\lambda 2}\\ \ldots \\ \partial \phi_{\lambda k} \end{array}\right)=\left(\begin{array}{c} J_{c1,\lambda 1}\\ J_{c1,\lambda 2}\\ \ldots \\ J_{c1,\lambda k} \end{array}\begin{array}{c} J_{c2,\lambda 1}\\ J_{c2,\lambda 2}\\ \ldots \\ J_{c2,\lambda k} \end{array}\begin{array}{c} J_{c3,\lambda 1}\\ J_{c3,\lambda 2}\\ \ldots \\ J_{c3,\lambda k} \end{array}\begin{array}{c} J_{a,\lambda 1}\\ J_{a,\lambda 2}\\ \ldots \\ J_{a,\lambda k} \end{array}\begin{array}{c} J_{b,\lambda 1}\\ J_{b,\lambda 2}\\ \ldots \\ J_{b,\lambda k} \end{array}\right)\left(\begin{array}{c} \partial c_{1}\\ \partial c_{2}\\ \partial c_{3}\\ \partial a\\ \partial b \end{array}\right).$$ (8)

The spectrally constrained reconstruction assumes the presence of a set of known chromophores and uses their spectral characteristics to constrain the solution space. This improves the accuracy of image reconstruction [17].

## 3. Results and discussion

### 3.1. Surface geometry imaging and mesh creation

**Fig. 2:**
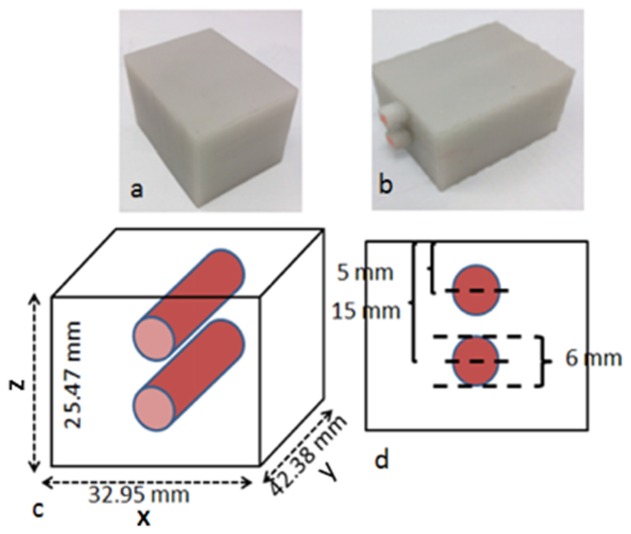
Plastic block phantoms. (a) homogeneous (b) heterogeneous with two absorbing rods inserted at the centre (c) and (d) the schematic diagram of the heterogeneous phantom.

The surface geometry imaging system is designed for imaging the hand especially in the area of the metacarpophalangeal (MCP) joint to provide data for the creation of the mesh that is required for image reconstruction. The system was firstly tested with a rectangular shaped block phantom that mimics the geometry of the third MCP joint, as shown in Fig. 2(a)

), to assess the performance of the current implementation of the system.

**Fig. 3:**
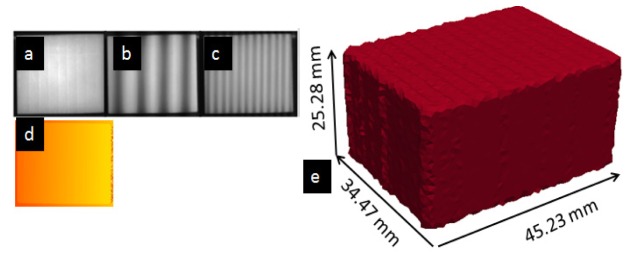
The surface geometry imaging of the block phantom. a) – c) the imaged projection of the fringe patterns at low, medium, and high frequency. d) The unwrapped phase map. e) mesh created from the surface geometry data.

Multiple frequency and phase-shifted fringe images were sequentially projected onto objects and were imaged using the system. Four frequencies at 1, 4, 20 and 70 waves per image were used with each frequency phase shifted at π/3, 2*π/3, pi, 4*π/3, and 5*π/3. Examples of the projected fringe image patterns at the first three frequencies are shown in Figs. 3(a)–3(c)

, with the unwrapped phase map illustrated in Fig. 3(d). The height map was firstly processed by applying a mean filter to reduce the sinusoidal artefacts. Prior to mesh generation, the height map was converted to a binary image volume by thresholding the intensity values. The x and y pixel dimension was retrieved using the calibrated camera model [23]. The accuracy was evaluated using the difference between the system-measured height and the calliper**-**measured nominal value. It was found that over 90% of the measured pixels of the height were within 0.4 mm, and over 85% of the pixels were within 0.3 mm, and over 60% of the pixels were within 0.2 mm with the root mean square error calculated as 0.2 mm. A height difference of less than 2 mm was found in lateral direction of the created mesh, as shown in Fig. 3 e), compared with nominal dimensions, as shown in Fig. 2(c). The accuracy of the height measurement was similar to previous works but with a shorter system acquisition time: 40 seconds versus 15 minutes and 4 minutes, respectively, for two recent systems [13, 20], as the number of fringe patterns utilised has been reduced.

**Fig. 4:**
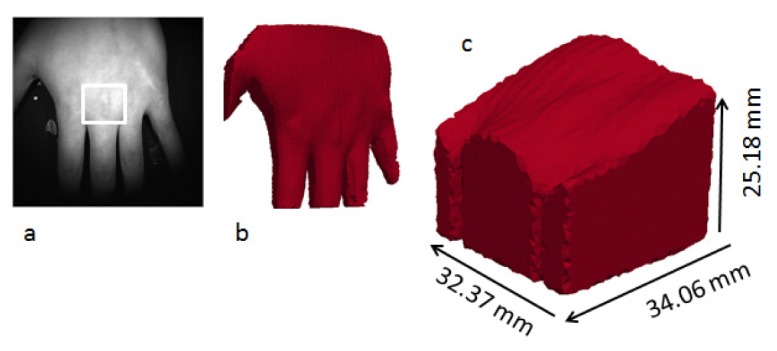
The surface geometry imaging of the whole hand. a) bright field image. b) whole hand mesh. c) cropped hand mesh to the third MCP joint as indicated as white frame in a).

The performance was further demonstrated with a realistic object, a male’s third metacarpophalangeal (MCP) joint. The whole hand image, as shown in Figs. 4(a) and 4(b)

, was cropped to a smaller region containing the third MCP joint to speed up the meshing process as well as the DOT image reconstruction. Figure 4(c) shows the mesh of the third MCP joint. The mesh points indicate a slope on the surface of the MCP joint with heights in a range from 20 to 30 mm. This was comparable with values measured using callipers, and the overall shape was in an agreement with the direct visualisation of the MCP point.

### 3.2. Spectral diffuse optical tomography imaging and reconstruction

#### 3.2.1. Simulation: the effect of source – detector number and system configuration

A simulation was used to evaluate the spectrally constrained reconstruction for obtaining chromophore concentration against a known modelled ground-truth. The real system configuration and imaging conditions, including the source-detector geometry and data type, were incorporated into the simulation to reveal the theoretical capability of the system and the optimal imaging conditions were extracted from additional simulations.

The boundary data from a mesh representing a heterogeneous block phantom, as shown in Fig. 2(b), was simulated using the forward model in NIRFAST. The mesh contains 28776 nodes and 159955 linear tetrahedral elements. The dimensions of the phantom are shown in Fig. 2(c) and the optical properties provided by the manufacturer are listed in Table 1

**Table 1:** Absorption and reduced scattering coefficients for the plastic block phantom.

**Background**		**Anomaly**	
**Absorption coefficients (mm^−1^)**	**Reduced scattering coefficients (mm^−1^)**	**Absorption coefficients (mm^−1^)**	**Reduced scattering coefficients (mm^−1^)**
0.0187 (690 nm)	1.1608	0.02 (730 nm)	1.8 (730 nm)
0.0179 (730 nm)	1.0662		
0.0181 (750 nm)	1.0648		
0.0162 (850 nm)	0.9825		
0.0149 (930 nm)	0.9515		

These values are provided by manufacturer (INO, Quebec, Canada).

. These absorption and reduced scattering coefficients were fitted to Eqs. (6) and (7) to obtain spectral parameters, such as the concentration of the absorbing dye, scattering amplitude and scattering power. The computation of concentration requires extinction coefficients of the homogeneous background to be known in advance which were are not available. In this instance, the homogenous background was assumed to have a ‘dye concentration’ of 1 (a normalise concentration to the background), and hence extinction coefficients were set equal to the absorption coefficients as listed in Table 1. The absorption coefficient for the anomaly was only given at a single wavelength (730 nm), and the normalised concentration with respect to the background was used, and its respective normalised concentration was calculated to be 1.068. The scattering amplitude and power were kept equal and constant for the background and anomaly regions as we were only interested in recovering the image contrast based on the absorption coefficients given that only CW data was used.

**Fig. 5:**
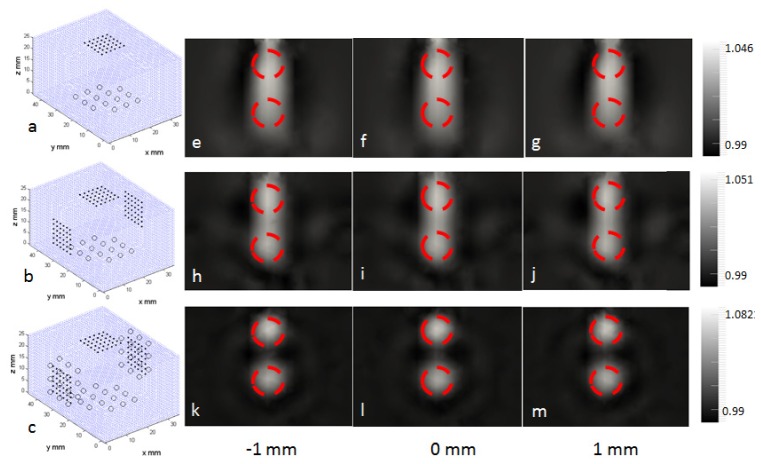
Image reconstructions using the simulated data. a) the source – detector geometry imposed by the system containing one set placed on the opposite side of the block. b) three sets of detectors placed in three orthogonal side with one set of source. c) three sets of sources and detectors placed on three orthogonal sides. e) – m) reconstructed images indicating the normalised concentration and anomaly regions for each source – detector geometry. Three slice through the y-axis of the volume are shown.

The first set of simulated boundary data was based on the source–detector geometry as shown in Fig. 5(a)

, corresponding to that used in our imaging system (see section 3.2.2 for details of defining the source – detector geometry for the system). There were 16 sources and 36 detectors with 2 sources omitted due to the low signal and the prolonged acquisition time. The sources and detectors had a regular grid layout with spacing of 5mm and 2.5mm respectively. In the reconstruction, to avoid the inverse crime, a second mesh that was the homogeneous version of this heterogeneous mesh was used. The initial normalised concentration used for image reconstruction was set as that of the background, as 1. The boundary data at 690 nm, 730 nm, 750 nm, 830 nm, and 930 nm were used simultaneously by applying the spectrally constrained reconstruction algorithm as discussed in section 2.3. The pixel basis in image reconstruction was set to the dimension of the block phantom to give a pixel size of 1 mm in each direction [26]. The initial regularization was set to 100 in Eq. (4). These values were selected based on the study summarised in section 3.2.3. The computation time was 2.2 hours for 12 iterations, and the speed of reconstruction was greatly depended on the mesh sizes. The reconstructed normalised concentration was 1.044 in the anomaly region which was within 2% of the target value. The two anomaly regions were however visually inseparable in the image. This used only a single imaging view with one array of source – detector pair, and two additional simulations were considered including an extended arrays of detectors, and extended arrays of both sources and detectors to investigate the possibility of resolving the two anomaly regions. These two additional source-detector geometries are shown in Figs. 5(b) and 5(c). The reconstruction from these simulations showed that the two anomaly regions could be correctly distinguished when sources and detectors were taken in all three orthogonal views, as illustrated in Figs. 5(k)–5(m), which could be practically difficult for current system configuration, whereas with only extended number of detectors the structure could still not be resolved, as illustrated in Figs. 5(h)–5(j). Quantitatively, with data taken in all three orthogonal views, the resolution has increased by 10 mm compared to that with data taken with the default source–detector geometry, whereas the contrast has been improved by 3.6%. The source-detector geometry imposed from the current imaging system indicated that not all structure was reconstructed, although optical property was well recovered.

#### 3.2.2. Phantom study I: solid phantom

**Fig. 6:**
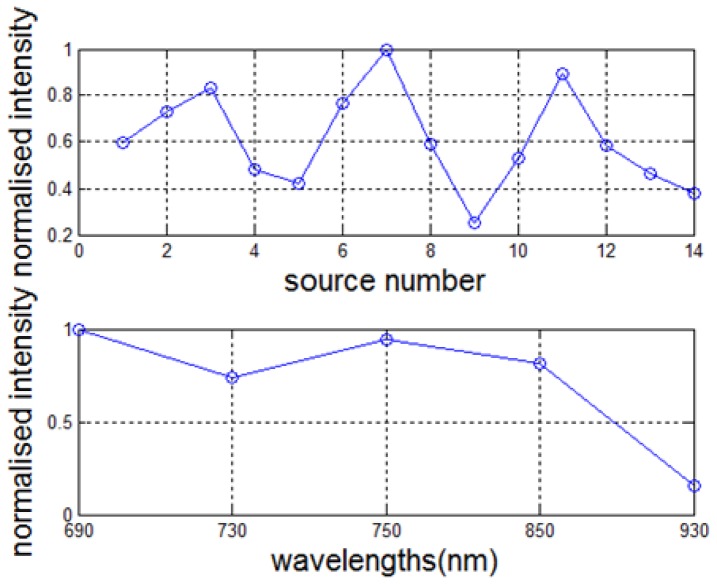
The system characteristic curve. (Top) The source strength variation curve: multiple light source illuminations at different positions are achieved using the butting coupling of optical fibres. Each of coupling associates with a coupling efficiency. (Bottom) The system spectral response: the system components including the camera and filters exhibits a characteristic spectrum.

The physical data measured from the system was used to demonstrate the system capability at a practical level. The heterogeneous block phantom was imaged on a wavelength-by-wavelength basis. The same source – detector geometry was used as in the simulation, as shown in Fig. 5(a)). The arrangement of virtual detectors was defined from the CCD camera image. Pixels in an 8x8 grid were grouped as one virtual detector, and the physical position of the virtual detector was retrieved using the camera model from which the physical size of each pixel was computed [23]. The source geometry of the system was found in a similar way by using the image pixel and the camera model. The raw data were normalised with the system characterization curve to account for the source strength variation and the system spectral response as shown in Fig. 6

**Fig. 7:**
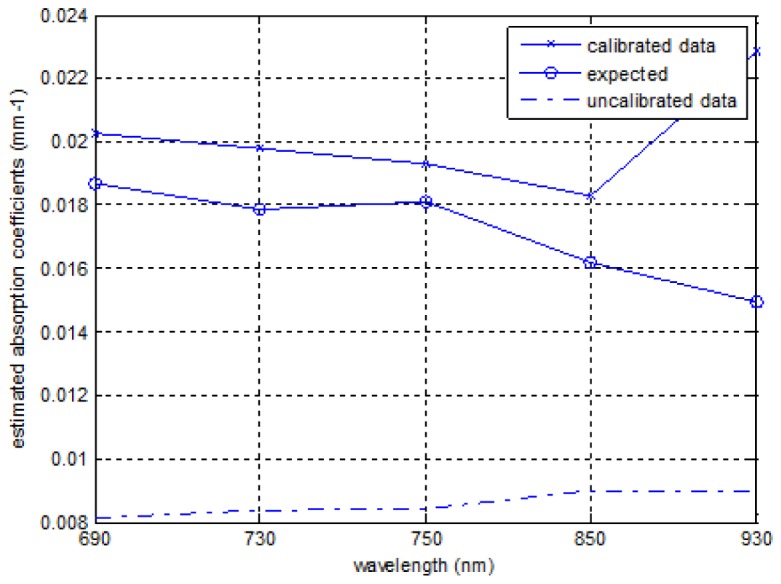
Comparison of calculated global absorption coefficients with manufacture provided values.

. The system spectral response was obtained by remounting the light source (unfiltered) in a reflection imaging geometry and illuminating a reflectance standard with 99% reflectivity (Labsphere, NH, USA), while the source strength variation curve was measured by imaging the source directly with a neutral density filter to prevent camera saturation. Alongside imaging of the heterogeneous phantom, the homogenous phantom was measured for calibration purpose that was essential for image reconstruction. The calibration step uses a homogeneous fitting algorithm to estimate the global optical properties [18]. These are used as an initial set of optical properties for image reconstruction and to compute the model offset which is calculated from the subtraction of simulated data (computed global absorption coefficients are used) from the system measured data. The calculated global absorption coefficients were found to give a comparable value to the manufacturer-provided values at 690 nm, 730 nm, 750 nm and 850 nm but a large difference at 930 nm, as shown in Fig. 7

. The data was thought to be noisy at 930 nm due to the quantum efficiency of the camera. The computed normalised concentration for the homogeneous phantom was 1.18, which was an 18% difference from the target value. A better estimate of the normalised concentration was 1.08 with only 8% deviation when the data at 930 nm was excluded.

**Fig. 8:**
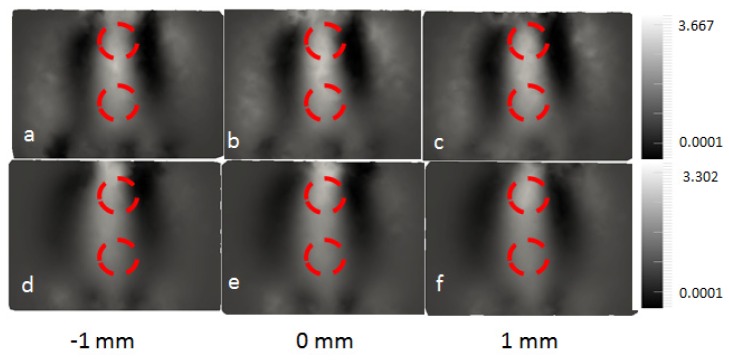
Image reconstructions using the physical data. a) –c) reconstructed using data of all five wavelengths. d)-f) reconstructed using data 930 nm excluded. Images show three slices from the centre of volume, at y = −1, 0 and 1 mm.

The calibrated heterogeneous data were used to reconstruct the internal distribution of normalised concentrations with respect to the background. As in the simulation, a homogeneous mesh was initially used together with the same pixel basis and the regularization constant applied. This homogeneous mesh was created using the captured surface geometry imaging data discussed earlier. The reconstructed images are shown in Figs. 8(a)–8(c)

. The two anomaly regions were again visually separated from the background but they were not distinguishable from each other. This agreed with the simulation study above, that the imaging geometry imposed by the proposed system limits the depth resolution of the system. The normalised concentration was found to have a peak value of 3.4 in the anomaly region and a peak value of 1.75 in the background. These values appeared to be different to the target value but the image contrast was preserved. This discrepancy suggests that the boundary data has some remaining offsets, and cannot be accounted for using the current calibration approach. This may also reflect a fundamental limitation due to scattering. The reconstruction was repeated with data at 930 nm excluded because of noisy data, as illustrated in Figs. 8(d)–8(f). The peak value of the normalised concentration is 3.3 at the anomaly regions, and is 1.19 at the background. As compared to utilising all data from all wavelengths, the contrast (between the background and the anomaly region) as reconstructed with four wavelengths data was increased by 46%, which was quantified by finding differences of peak values of the normalised concentrations.

#### 3.2.3. Phantom study II: liquid phantoms

**Fig. 9:**
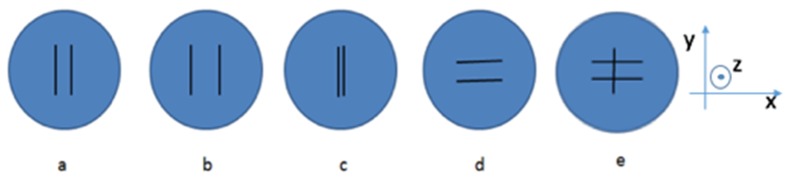
Petri dish milk phantoms. a) two parallel rods separated at 11 mm. b) two parallel rods separated at 15 mm. c) two parallel rods separated at 4 mm. d) rotated 90 degree of a). e) Two parallel rods separated at 15 mm are suspended on an additional rod lying above forming a compound structure.

A selection of different phantoms was used to demonstrate the system capability and to reveal additional performance information of the system. The results are described in terms of evaluating the system on data from imaging five liquid phantoms in this section. The schematic diagrams of these liquid phantoms are shown in Fig. 9

. Each phantom consists of a petri dish of 84 mm in diameter and 14 mm in depth with plastic rods fixed at a depth of 7 mm in the petri dish. The rods are acting as an absorbing anomaly in a homogeneous background composed of semi–skimmed milk. The rods were embedded in different arrangements in each case, in addition to a homogeneous reference milk phantom which was used for calibration purpose. The liquid phantoms were placed in the centre of the field of view so that the centres of the phantoms were illuminated. Only these areas were considered to avoid the need to move the phantom. The global bulk optical property was fitted using the homogeneous fitting algorithm as described above, and the normalised milk concentration was assumed to be 1 in the background. The reconstruction was effectively to obtain the normalised concentration, of the milk in the region where the rods were, with respect to the background to assess the lateral resolution of the system.

**Fig. 10:**
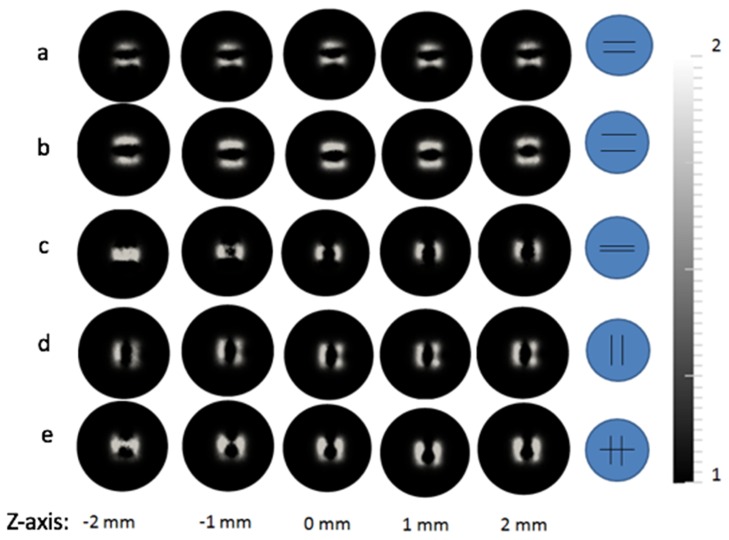
Reconstructed liquid phantoms: a) two parallel rods separated at 11 mm. b) two parallel rods separated at 15 mm. c) two parallel rods separated at 4 mm. d) rotated 90 degree of a). e) Two parallel rods separated at 15 mm are suspended on an additional rod lying above forming a compound structure.

The source–detector geometry was defined as in the earlier experiments, and contained 49 detectors with a spacing of 2.5 mm in a regular grid layout. The same source geometry as in Fig. 5(a) was used. The pixel basis was set to the same value as before, and the initial regularization was set to 100. These reconstruction parameters were selected based on evaluating the accuracy of rod separation by measuring the line profile in reconstructed images, by using a wide range of these values. The reconstruction results for all five phantoms are shown in Fig. 10

. For each one, five slices extracted from +/2 mm each side of centre of volume are shown. From these images, the phantom with a rod separation of 11 mm, a rod separation of 15 mm and a rotated rod separation of 11 mm, as shown in Figs. 10(a), 10(b) and 10(d), were reconstructed as expected. Figure 10(c) shows that the reconstruction cannot resolve rods placed 4 mm apart. In the reconstructed images of the phantom with the compound structure, as shown in the Fig. 10(e), the structure is only visible in one slice. For all liquid phantoms, the normalised concentration in the background, which was 1, showed a good agreement to the target values. The target value in portion of milk (normalised concentration) in the rod regions were not known so their quantitative accuracy could not be justified, but a high value, 2, was shown in these regions due to strong attenuations.

### 3.3. Imaging hand joints with ultrasound image co-acquisition

**Fig. 11:**
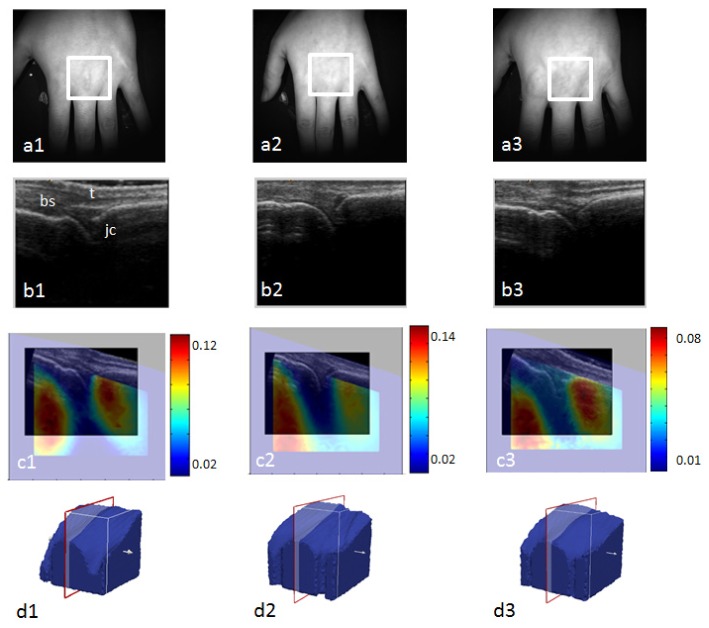
Reconstructed images of the HbT concentration (unit: milli molar) in the third MCP joint from three volunteers, overlapped on the ultrasound image co acquired on the same location. a1) – a3) the bright field image for each volunteer. the region used for DOT reconstruction as indicated by the white frame. b1) – b3) the co acquisition of ultrasound image. bs: bone surface; t: tendon; jc: joint cavity. c1) – c3) registered DOT images and ultrasound images. d1) – d3) the position of the DOT image and ultrasound image slice position for each volunteer.

The results presented in this section are based on data measured from three healthy volunteers using the system. Healthy volunteers were first imaged with ultrasound to act as a reference for comparison. The left third (MCP) joints were scanned and sonography identified the joint's anatomical structure including bone surface, tendon and joint cavity, as shown in Fig. 11(b1)

. The volunteers then underwent spectral DOT imaging, and the ultrasound scanning locations were manually aligned with source positions in the DOT system. Figure 11(a1)–11(a3) shows the bright field images of the subjects’ hands. DOT data was measured from the volume where the ultrasound image was acquired, and the volume was centred with this ultrasound image. The white frames in Fig. 11(a1)–11(a3) highlight the region of the interest. For this instance, measurements of the homogeneous block phantom were used for calibration purpose. The global optical properties were calculated using the homogeneous fitting algorithm directly on the DOT data from imaging the MCP joint, and used as the initial values for the image reconstruction. The mesh used for reconstruction was initially homogeneous and was generated using the surface geometry imaging data from each volunteer. The detectors were in a regular grid layout with a spacing of 4 mm, and the source geometry was the same as in Fig. 5(a). For image reconstruction, the pixel basis was set so that each dimension of the joint mesh had an mm resolution. The initial regularisation was set to 100, and image reconstruction time was typically around 20 minutes using a PC with an Intel Core 2 quad CPU Q8200 at 2.33 GHZ and 8 GB RAM. The recovery of water concentration and data at 930 nm were excluded from reconstruction as the data was found to be noisy and the water absorption coefficient high at this wavelength.

Figures 11(c1)–11(c3) shows the reconstructed images indicating the total hemoglobin concentration (HbT) overlaid with the ultrasound (US) images taken at the same location for three volunteers. The DOT image is shown using the “jet” colour map, while the US image is shown in grayscale. Figures 11(d1)–11(d3) indicates the DOT image slice orientations for these images in Figs. 11(c1)–11(c3) for three volunteers with the red frame drawn around the volume. The boundary of both ultrasound and DOT images was removed to assist the visualisation; these regions were primarily dark pixels and boundary artefacts. It appears in the DOT images that the recovered HbT is lowest in the centre of image, and is the highest at the sides, which are 10%, 13.8% and 7% larger compared to the centre for each volunteer, respectively. In reference to the registered US image, the drop in HbT in the centre of the images is found to correspond to the joint cavity, and the elevated HbT at the edges corresponds to the bone region. These image features matched with those from the previous work that showed the healthy joint had a reduction in absorption coefficients in the joint cavity as compared with surroundings [9]. According to Eq. (6), the absorption coefficient is linearly proportional to the chromophore concentration, and a low absorption coefficient region would be expected to have a low chromophore concentration, in this case, low HbT. This is thought to be due to the fact that the joint cavity is filled with a clear fluid that is weakly attenuating. The spectral DOT image consequently reveals low HbT in this region compared to surrounding tissue that has embedded vasculature and microcirculation containing blood. A similar image feature was observable for all three data sets.

## 4. Conclusion and future work

Diffuse optical tomography has the potential and promise to provide diagnostic value in the early stage of rheumatoid arthritis. A multi-wavelength diffuse optical tomography system has been developed and presented in which data at multiple wavelengths has been incorporated simultaneously in a spectrally constrained reconstruction algorithm, with the surface geometry imaging method integrated to directly extract the distribution of physiology relevant parameters in the hand joint. The system has been demonstrated with simulations, phantoms, and on human hand joints.

It has been shown that by imaging the rectangular block phantom and a hand on the third MCP joints, the system produced accurate surface geometry data and is capable of capturing the whole hand surface geometry. The surface capture component can be optimized in future in several ways, for example, using a high frame rate camera and accessible projector for ultimate speed and automation control.

A simulation study has been presented in which the 3D rectangular block phantom together with two anomaly regions was modelled. With simulated spectral data, the normalised concentration was reliably recovered using a spectrally constrained reconstruction algorithm. However, the expected anomaly regions were not visually distinguishable in reconstructed images. The comparison with two additional simulations considering two different source–detector geometries, which includes an extended view of detectors and an extended view of both detectors and sources, suggested that the source–detector geometry, a single view, imposed by the system limited the resolution of two anomalies. The image contrast based on the dye concentration recovered was promising, and some useful structural information was also obtainable.

Reconstructed images from imaging the block phantom and the liquid phantom study were presented in which the physical data including surface geometry and boundary measurements were obtained from the system. For the block phantom, the reconstructed image feature was similar to the simulation carried out in the earlier study that the anomaly regions were highlighted from the background but the two regions were virtually unresolvable from each other. The quantitative recovery of concentration was not as good as compared with the simulation. From the study with a number of liquid milk phantoms, it shown that the system was unable to resolve a 4 mm feature with optimal imaging conditions, such as the detector spacing, and reconstruction parameters. The camera binning and the size of virtual detector were primarily found to affect the accuracy and the theoretical validity of the boundary measurement, and these may allow the quantitative accuracy of reconstruction to be improved in future. Although the system was not able to resolve all features accurately, we note that the study was based on phantoms with much stronger absorbers than biological tissues, which scattering events dominated.

Reconstructed images from imaging the left hand third MCP joints of three healthy volunteers have been presented. The images of HbT showed a prominent feature that corresponded well with previous work. A drop in HbT was found in the centre of the image in the area of the joint cavity suggesting that the reconstructed images of HbT are consistent with known physiology. The overlay of ultrasound data revealed good correspondence of the joint region with reconstructed HbT images [9]. Although this work focused only on recovering the value of HbT for healthy subjects, it is expected that other recoverable parameters, such as oxygen saturation, water content and scattering parameters may provide additional information for assisting RA diagnosis, which will be a subject of future studies. The DOT acquisition time was in the range of 5 – 8 minutes depending on the thickness of the joint which is longer than a previously reported system [13]. A few improvements have been immediately noted to potentially reduce the acquisition time: 1. Switching between filters could be optimized. 2. The number of sources used for illumination may be more than is required. 3. A larger virtual detector would reduce the acquisition time. The main advantage of the presented system is the non-contact nature of the measurement strategy and although the results demonstrate limited depth resolution, the measured contrast of recovered optical properties through spectral measurements provide a consistent and stable data as compared to existing single wavelength, full tomographic systems, which is again demonstrated here through phantom and healthy subject studies.

There exist more recent developments in instrumentation and data collection strategies, such as the use of structured illumination through Time-Resolved data which can further enhance resolution and contrast of diffuse optical tomography [27]. These advancements shows great improvement over standard techniques, which hold promises for further application of diffuse optical tomography in RA imaging, specifically with applications to whole hand imaging.

In summary, the developed system can image and reconstruct image contrast based on absorption coefficients, and produce spectral information relating to the concentration of chromophores, although the system configuration and the quantitative recovery of optical properties could still be improved further. One noticeable result was that we showed image contrast consistency with previous studies corresponding to the hand joint region. Future work includes system optimization (especially quantitative reconstruction and acquisition time), and imaging of arthritic joints with a sufficiently large sample to provide a thorough validation of the system to support clinical use.
